# Domain affiliated distilled knowledge transfer for improved convergence of Ph-negative MPN identifier

**DOI:** 10.1371/journal.pone.0303541

**Published:** 2024-09-27

**Authors:** Md Tanzim Reza, Md. Golam Rabiul Alam, Rafeed Rahman, Shakib Mahmud Dipto

**Affiliations:** 1 BRAC University, Dhaka, Bangladesh; 2 University of Liberal Arts Bangladesh, Dhaka, Bangladesh; Universidade Lisboa, Instituto superior Técnico, PORTUGAL

## Abstract

Ph-negative Myeloproliferative Neoplasm is a rare yet dangerous disease that can turn into more severe forms of disorders later on. Clinical diagnosis of the disease exists but often requires collecting multiple types of pathologies which can be tedious and time-consuming. Meanwhile, studies on deep learning-based research are rare and often need to rely on a small amount of pathological data due to the rarity of the disease. In addition, the existing research works do not address the data scarcity issue apart from using common techniques like data augmentation, which leaves room for performance improvement. To tackle the issue, the proposed research aims to utilize distilled knowledge learned from a larger dataset to boost the performance of a lightweight model trained on a small MPN dataset. Firstly, a 50-layer ResNet model is trained on a large lymph node image dataset of 3,27,680 images, followed by the trained knowledge being distilled to a small 4-layer CNN model. Afterward, the CNN model is initialized with the pre-trained weights to further train on a small MPN dataset of 300 images. Empirical analysis showcases that the CNN with distilled knowledge achieves 97% accuracy compared to 89.67% accuracy achieved by a clone CNN trained from scratch. The distilled knowledge transfer approach also proves to be more effective than more simple data scarcity handling approaches such as augmentation and manual feature extraction. Overall, the research affirms the effectiveness of transferring distilled knowledge to address the data scarcity issue and achieves better convergence when training on a Ph-Negative MPN image dataset with a lightweight model.

## 1 Introduction

Ph-negative Myeloproliferative Neoplasm (MPN) is a hematological disorder that is caused due to JAK2, CALR, and MPL mutation [1, 2] which drives up the proliferation of blood cells in the bone marrow [3]. The resultant symptoms are headaches, splenomegaly, abdominal issues, fatigue, fever, and in severe cases, it sometimes turns into Arterial or venous thrombosis that occurs in the form of ischemic stroke and other adverse cardiovascular events. The thrombosis is caused by a higher than usual count of hematocrit and white blood cells during the occurring period of the disease, with the probability of the incident being boosted by clinical factors such as obesity, older age, hypertension, dyslipidemia, and previous thrombosis occurrences. Aside from thrombosis, the patients can also develop more severe symptoms like leukemia, which can ultimately impact the mortality of the patients [4].

Ph-negetive MPN is considered rare due to only 6 per 100,000 people being affected by it every year [2]. While the disease itself rarely causes death, it is still dangerous due to its nature of turning into fatal cancers such as leukemia and other severe cases [5]. Research conducted by the Swedish Cancer Register claimed that the mortality rate of adult MPN-affected patients can be attributed to hematological malignancies and bacterial infections, while young patients typically die from cardiovascular diseases [6]. Therefore, timely prognosis and treatment of the disease are essential. For proper prognosis, the disease needs to be identified and distinguished by three different subtypes; namely Essential Thrombocythemia (ET), Polycythemia Vera (PV), and Idiopathic Myelofibrosis (MF) [7].

For clinical diagnosis of Ph-negetive MPN, laboratory-based screening of JAK2 mutation is performed despite being slightly unreliable due to false-positive or false-negative test results, especially in patients with ET and MF subcategories where the effect of the mutation is often observed to be missing [8, 9]. Therefore, bone marrow morphological examination is required in addition to the mutation screening for a confirmed diagnosis of ET and MF, where the presence of abnormal mast cells is used as an indicator of subtype positive [10]. Meanwhile, work on the application of an Artificial Intelligence (AI) based automated diagnosis system started in 2002 [11] and so far the field has been dominated by handcrafted feature extraction procedure for the diagnosis of the disease subtypes [11–14]. In contrast, the history of Deep Learning (DL) based applications has been relatively limited, with only the presence of some contemporary experiments [15–18]. The below proportionate ratio of DL-related research can be attributed to the lack of available pathological data. As DL model training is mostly data-driven and a large number of samples is required to obtain satisfactory results, achieving a well-functioning model can be arduous as training samples are difficult to come by because of the rarity of the disease. The source of pathological image samples is rare, and the existing sources have a rather limited number of samples [19].

The proposed research strives to make maximum use of the limited available Ph-negative MPN pathological samples by utilizing distilled knowledge from a much larger histopathological image collection. To achieve this, a teacher model is trained on a large collection of histopathologic scans of lymph node sections, followed by the training knowledge being distilled to a smaller student model. Afterward, the student model is separately trained to identify Ph-negetive MPN subtype from a smaller dataset. Empirical analysis showcases that the student model with distilled knowledge achieves significantly improved convergence compared to a clone model trained from scratch. While the overall training pipeline is more sophisticated than other small dataset handling procedures such as augmentation, the resultant outcomes are generally better. The contributions of the proposed research work are:

- We have introduced a framework specifically designed to perform well for Ph-negetive MPN identification despite the histopathological image scarcity issues, contributing to the limited field of DL-based research on the disease.
- We have designed a knowledge distillation-driven pipeline that transfers domain-specific knowledge from a large histopathological image dataset. The pipeline gains maximum benefit by training a large model on the bigger dataset, followed by the knowledge being distilled to a smaller model for lightweight end-user deployment.
- We have provided a comprehensive empirical analysis to prove the reliability and the viability of the procedure in comparison to other low-resource data handlers competitors such as augmentation and handcrafted feature extraction. Comparing the proposed approach to other similar ones, at least 4–5% performance boost is achieved under a similar parameter setup.
- We have demonstrated the superior performance of the presented distillation pipeline over traditional transfer learning, showcasing the effectiveness of utilizing domain-affiliated knowledge.

The rest of this paper is divided into a background study in chapter two, proposed methodology in chapter three, result & analysis in chapter four, and a conclusion in chapter five, respectively.

## 2 Literature review

The literature review consists of details on Ph-Negetive MPN subtypes, contributions of the existing SOTA literature, and identification of the research gaps. The following sections consist of all the learned details.

### 2.1 Ph-negetive MPN subtypes

As mentioned previously, Ph-negative MPN can be divided into three subtypes: PV, ET, and MF. A brief description of the subtypes based on WHO’s diagnostic criteria [20, 21] is provided below.

**Polycythemia Vera (PV)**—A higher concentration of erythrocyte and thrombocyte, alongside a reduced production of Endogenous erythropoietin hormone often signals the presence of PV. JAK2 V617F mutation test almost always returns a positive result for this subtype. In terms of risk factors, PV creates an increased risk of myelofibrosis and thrombosis.

**Essential Thrombocythemia (ET)**—Morphological features of ET are represented by enlarged megakaryocytes, a precursor cell that forms and releases thrombocytes in the bloodstream. This causes an increased density of thrombocytes in the blood. JAK2 mutation test is often not enough for a proper diagnosis of ET, additional bone marrow morphological tests are required. Similar to PV, ET causes an increased risk of myelofibrosis and thrombosis, which may progress to other MPN subtypes depending on the mutation type.

**Idiopathic Myelofibrosis (MF)**—Among the three subtypes of Ph-Negetive MPNs, MF is the most aggressive one with severe symptoms. MF increases the quantity of Granulocytic and megakaryocytic in the bone marrow, alongside causing various abnormalities in the peripheral blood. Akin to ET, bonemarrow morphological tests are also required alongside JAK2 mutation test to confirm the occurrence of the subtype. Depending on the severity of the subtype, it may progress to acute myeloid leukemia.

### 2.2 Existing works

The idea of the automated diagnosis of Ph-Negetive MPN started back in 2002 as the initial phase has been dominated by handcrafted and lab experimentation-based feature processing. To diagnose PV, Kantardzic et al. [11] extracted four important features from lab experiments namely Hematocrit (Volume of Red Blood Cells), Platelet Count, Splenomegaly (Enlargement of Spleen), White Blood Cell count, and then applied a rule-based threshold to convert them to categorical features. Afterward, they trained a Neural Network model alongside an SVM model, and on the four feature sets, the models achieved 98.1% accuracy and 95% accuracy, respectively.

There have also been dedicated works on subtype classification based on clinical features. Korfiatis et al. [12] collected clinical data based on the concentration of hemoglobin, white blood cells, red blood cells, and platelets. In addition, data based on sex, age, kidney ultrasound, liver ultrasound, hematocrit, and JAK2 mutation test were collected. Furthermore, two features for blood pressure measurement and three binary features for the existence of other diseases were added. In total, 15 different features were collected for the diagnosis of primary and secondary polycythemia, which is a parent category of PV. The classification was performed at two levels: at the first level, input data was classified into healthy and non-healthy types while at the second level, the existence of primary and secondary polycythemia was classified from the non-healthy type. The authors used LM-FM, a wrapper feature selection algorithm, to try different subsets of the initial 15 features to train an SVM model. Using a subset size of five at each level, they achieved 98.9% accuracy on the first level, and 96.6% accuracy on the second level. The first level used the age, hemoglobin concentration, red blood cell concentration, JAK2 mutation, and liver ultrasound while the second level used sex, white blood cell concentration, JAK2 mutation, kidney ultrasound, and 2nd other disease subtype features. Although the performance was extremely good, collecting so many features for classification can be difficult.

Z. E. Fitri and A. M. N. Imron [14] classified WBC abnormalities from peripheral blood smear images of ET-affected patients. At first, the images went through color conversion, WBC cell segmentation, and afterward, morphological features such as area, perimeter, metric, and compactness of WBC were extracted from the images. Using the four morphological features they achieved 91.82% test accuracy with a Multi-layer Perceptron model. While the overall performance was good, the authors mentioned that the morphological feature values were close to each other for the normal and abnormal classes, which impacted the final score. To improve the performance, additional feature types such as textures could be added. Meanwhile, Meggendorfer et al. [22] proposed a system to differentiate subtypes of MPNs by combining Complete Blood Count parameters and genetic information using a Next Generation Sequencing panel consisting of 18 genes. They achieved an accuracy of 100% in PV detection, and 88% in ET detection. However, the system faced a hard time classifying MF as only 21% accuracy was achieved.

Machine learning is also used for the segmentation of cells that aid in MPN detection. T. H. Song and his co-researches [13] segmented Megakaryocytic cells, which play a crucial role in the diagnosis of Ph-Negetive MPNs, from bone marrow images. They picked color and texture features to use on a dual-channel active contour model to separate Megakaryocytic cells from the cytoplasm. The authors claimed that their novel approach achieved a precision score of 0.86 and a recall score of 0.833 in the task of finding the nuclei of the Megakaryocytic cells.

Deep learning-based diagnosis from histopathological images is the recent advancement in the field of MPN classification. Kimura et al. [18] combined a complete blood cell count and CNN-extracted morphological deep features from pathological images to differentiate between PV, ET, and MF. Overall, they achieved sensitivity scores of 100%, 90.6%, and 100% respectively while the specificity scores were 95.4, 95.2, and 90.3%, respectively.

Yusof et al. have done extensive research in Ph-Negetive MPN subtype classification. In one research, the authors classified between PV, ET, and MF using a 3-layer CNN feature extractor attached to a 3-layer MLP classifier. In the process, the dataset was cleaned, augmented, and using the CNN model, the authors experimented with 10-fold cross-validation using various types of input shapes, batch sizes, and optimizers. They figured out that the model provides the best output on an input size of 64x64, batch size of 100, and adamax optimizer with an accuracy of 94.6%. The final outcome was an accuracy of 95.3% on a select test set, with the highest recall value of 96.46% on ET, followed by 94.95% on MF, and 94.44% on PV [15].

In another study [16], to classify MPN subtypes, the author constructed two datasets, one is a control dataset of original bonemarrow histopathological images, while the other one is a reconstructed pathology imagery dataset from a Super Resolution CNN. Both datasets consisted of 600 images, 200 images for each of the three MPN subtypes. After cross-validation and hyperparameter tuning, the authors achieved an improved accuracy of 92% on the reconstructed image dataset, compared to the 88% accuracy on the original dataset. This particular work was preceded by other research [17] where the authors only worked on hyperparameter selection for deep learning-based classification of the three MPN subtypes. In their work, after hyperparameter tuning on a CNN, they achieved an accuracy of 91.64% with 32x32 input image size, 120 epochs, and RMSprop optimizer.

Finally, Yusof et al. contributed further by releasing an open-source dataset on histopathological images of MPN subtypes [19]. The dataset consists of 300 images of MPN disease-associated bone marrow trephine, with 100 images for each of the subtypes. The authors addressed the issue of the dataset being small in terms of sample size with the fact that the MPN disease has rare occurrences, a claim that was also made in various other research and review papers [23–25]. Despite the small sample size of the dataset proposed in [19], it is still one of the most relevant datasets in terms of histopathological image collection of the disease and thus, we have decided to use it in our research.

The possibility of enhancing a smaller student model’s performance with the aid of a larger teacher model gave rise to the concept of knowledge distillation. The knowledge distillation process may accomplish variants of tasks, including fusing features from different intermediate layers to improve the performance of another model [26], compressing a larger model to a smaller model for reducing memory usage and improving inference speed [27, 28].

To utilize the method of knowledge distillation, Shengyuan Tan and his co-researchers extracted features from different portions of a teacher and a student network, and performed feature correction utilizing a Squeeze-and-Excitation block. After upsampling the lower-dimensional features and downsampling the higher-dimensional features, features from several layers were fused. The fused feature map seemingly contained more information than the individual feature maps and helped the student model to perform better [26]. Jeon et al. utilized a transformer compression technology to reduce the size of both the encoder and decoder, as opposed to the usual process of reducing just the size of the encoder [27]. Meanwhile, Kim et al. compressed graph convolutional networks by utilizing the final hidden embeddings alongside the task predictions. Ultimately, the general purpose of these experiments are same, compressing models without losing much of the performance through utilizing knowledge of the larger model [28].

As medical image databases are frequently limited and come in several modalities for the same disease and patient, the use of knowledge distillation extends greatly to the application of disease classification. Consequently, knowledge distillation can be utilized to make the most out of small datasets or transfer knowledge from one modality to another modality. For example, few researchers fuse knowledge from CT and MRI modalities for improved performance on one of the modalities in terms of classification or segmentation [29–31]. In such cases, the teacher model usually learns from both modalities, and the knowledge is distilled to the student for performance improvement on individual modalities.

Histopathological image-based analysis can also benefit from the application of knowledge distillation. Pathologists have been identifying various diseases by examining tissue samples on slide glass. Histopathological images are created by scanning the whole slide and forming a digital image [32] which can be used to classify a vast range of cancer-related diseases such as Lymphoma, breast cancer, colorectal cancer, ovarian cancer, and many more. Although the images for different diseases can show slight variety in terms of visuals due to different levels of magnification while scanning, different colors, and variations of artifact [33], the general visual structure is similar. Thus, there is ample scope for transferring knowledge earned from image databases of one disease to process image databases of another disease.

Upon reading the previous research work, our overall finding is that histopathological image-based DL training is very rare for MPN classification, with only a few research papers available at the given time. Most of the existing research papers rely on using traditional ML algorithms on various clinical features. Although this can yield great results, collecting so many different types of clinical feature data can be a troublesome process. Even in the case of the existing machine learning based works, the utilized histopathological datasets are very small because the disease is rare. In addition, in the majority of the works, the authors either worked on classifying a particular MPN subtype or achieved poor performance on one of the subtype classifications when training was done on all three major subtype samples. Knowledge distillation, in the meantime, shows good promise for histopathological image analysis. Therefore, we concisely worked on addressing the existing issues based on our distillation-driven model.

### 2.3 Knowledge distillation

As mentioned previously, knowledge Distillation [34] is the process of transferring learned knowledge from a larger model to a smaller computationally efficient model. It can reduce the consumption of computational resources while maintaining the performance, sometimes even improving it. Generally, the larger model is referred to as the teacher model while the smaller model is referred to as the student model. In a distillation environment, the teacher model learns from the data first, followed by the student model learning from both the teacher and the dataset in the next step. During the student training session, the student model tries to replicate the learned knowledge of the teacher. When utilized on a distillation pipeline, the student model can generally perform better than when trained from scratch.

## 3 Methodology

**Fig 1:**
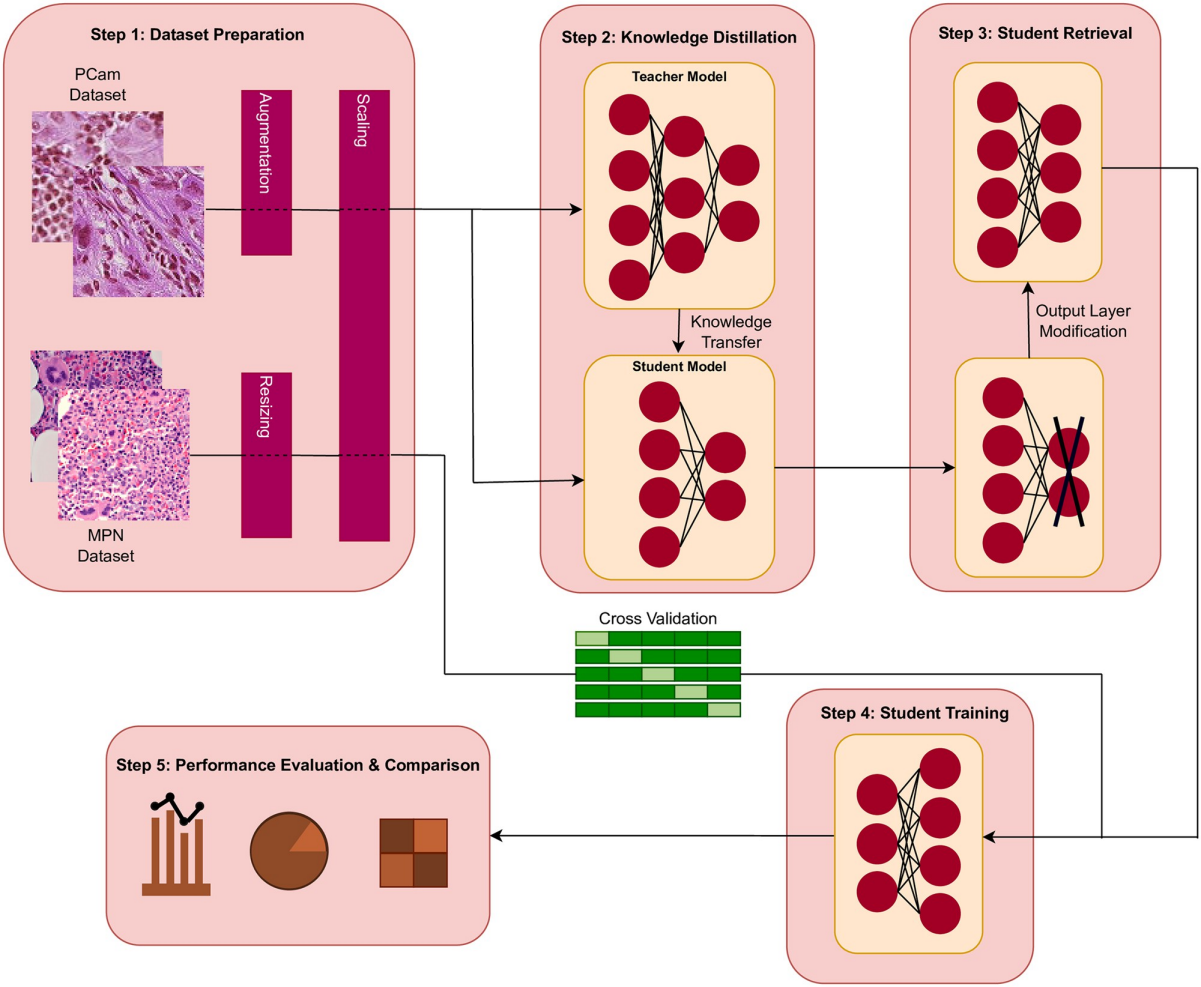
Symbolic workflow of the main training pipeline.

The pipeline of the proposed research work is given in Fig 1 which is primarily divided into five steps, starting from dataset preparation to knowledge distillation, loading the distilled model, further training it on the MPN dataset, and finally performance evaluation against other approaches. The following few subsections discuss the step-by-step approach.

### 3.1 Step 1: Dataset preparation

#### 3.1.1 Dataset description

**Fig 2:**
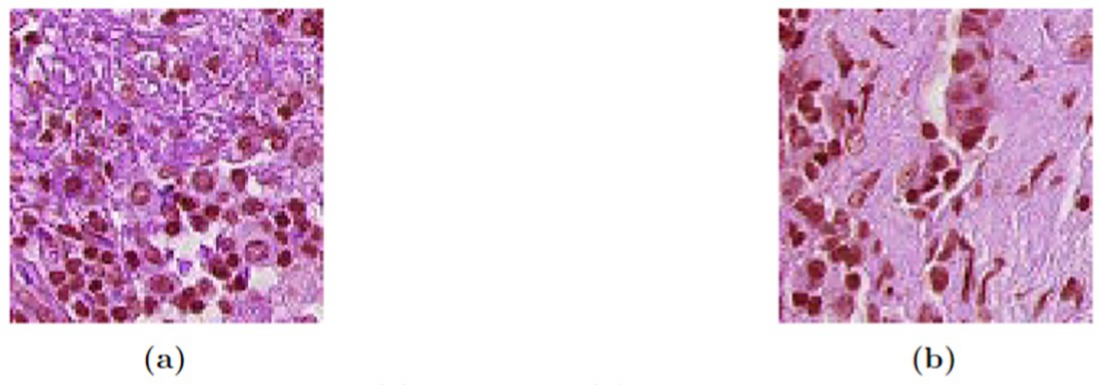
PCam dataset classes. (a) No Tumor (b) Tumor.

The proposed methodology comprises the usage of two publicly available datasets, one is a large dataset named PatchCamelyon (PCam) [35] and the other is our target MPN dataset [19], which is much smaller in size. The PCam dataset consists of 327,680 RGB image samples of 96x96 resolution which visualize the histopathologic imagery of lymph node sections. This is one of the largest collections of histopathological images and is a great choice for learning generalized patterns. The dataset is labeled by two classes: a positive label which indicates the existence of a tumor tissue in the center region, and a negative label which indicates the absence thereof. The dataset comes with a pre-distributed train-test-validation set. Table 1 entails the overall sample distribution of the dataset, while Fig 2 shows some sample images from the PCam dataset classes.

**Table 1:** PCam dataset sample distribution.

	Train	Validation	Test
**0-Without Tumor**	1,31,072	16,399	16,391
**1-With Tumor**	1,31,072	16,369	16.377
**Total**	2,62,144	32,768	32,768

**Fig 3:**
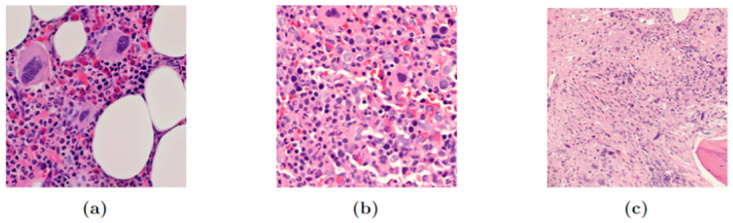
MPN subtype samples. (a) ET (b) PV (c) MF.

Meanwhile, the MPN dataset is a small collection of sample images of Ph-Negative MPN subtypes, with only 100 images of each of the classes. The data was collected from Hospital Serdang, Malaysia with proper ethical approval granted by the Ministry of Health, Malaysia. The resolution of the images varies ranging from a minimum of 927 × 927 pixels to a maximum of 2036 × 2036 pixels. Some of the samples from the MPN dataset are given in Fig 3, while the sample distribution is given in Table 2.

**Table 2:** Ph-negative MPN dataset class distribution.

	Number of Images
**ET**	100
**MF**	100
**PV**	100
**Total**	300

The combination of two datasets is a good setup for the knowledge distillation environment as both being histopathological imagery of bones, they share lots of similar spatial features. providing a good platform for knowledge transfer from one dataset to another. Additionally, learning generalized knowledge, even if it is domain-specific, requires access to lots of data. Since PCam dataset has a large number of samples, it serves as a good source for generalized learning, especially when a large eligible model is used for training. Therefore, we selected the PCam dataset for transferring knowledge to the training on the MPN dataset.

#### 3.1.2 Data pre-processing

Before training, both datasets went through some dedicated pre-processing techniques. In the distillation environment, the PCam dataset went through slight augmentation and pixel value normalization within a range of 0 to 1, where the pixel values were normalized through a division of 255. Despite the PCam dataset being very large, some primary-level augmentation was still performed such as width shift, height shift, and horizontal flips. The default resolution of the PCam dataset is 96 × 96, far lower than the resolution range of the MPN dataset. Consequently, to match the resolution and input size of the model across different stages of the training, we resized the images of the MPN dataset to 96 × 96. The details of the processing steps are given in Table 3.

**Table 3:** Processing steps.

Processing type	Applied on (Dataset)	Details
**Normalization/Scaling**	PCam, MPN	0–1 Range
**Augmentation**	PCam	10% width shift, 10% height shift, Horizontal flip
**Resizing**	MPN	To 96 × 96 resolution

### 3.2 Step 2: Knowledge distillation

The distillation setup involves the PCam dataset, the teacher model, and the student model. For the teacher model, a 50-layer ResNet [36] model which was pre-trained on the ImageNet [37] dataset was used. We intentionally used a large-scale pre-trained model as the teacher model to get the maximum learning from the larger dataset. For the student model, since the target MPN dataset is extremely small, we opted for a very lightweight 4-layer CNN model so that the model does not overfit the target dataset.

#### 3.2.1 Teacher model (ResNet)

The ResNet model is an established CNN architecture that was first proposed in 2016. The specialty of the ResNet model lies in the usage of residual learning, which uses a form of skip connection. CNN models usually benefit from having deeper architectures, as the deeper layers can generally extract more fine-tuned morphological features such as shapes. However, making a CNN too deep introduces the vanishing gradient [38] issue, where the gradient becomes extremely small as they backpropagate to the earlier layers of a CNN, resulting in very small to zero learning in the earlier layers. The ResNet model tackles this issue by introducing skip connections where the gradient can directly backpropagate through the skips without any modification. Due to the presence of this skip connection, some parts of the gradient can flow to the earlier layers with little interruptions and the earlier layers’ weights can be updated. ResNet architectures come in various depths, but the most commonly used ones are 50-layer, 101-layer, and 152-layer deep ResNet. The higher-depth ResNets are usually harder to train and are for very large datasets, so we opted to use the 50-layer deep variant. We removed the existing classifier of the ResNet and added custom classifier layers consisting of a fully connected layer of 1024 neurons followed by the output layer of two neurons. The layer with the 1024 neuron uses ReLU activation while the output layer uses softmax activation. The model was compiled with categorical cross-entropy loss and Adam optimizer.

#### 3.2.2 Student model (4-Layer CNN)

**Fig 4:**
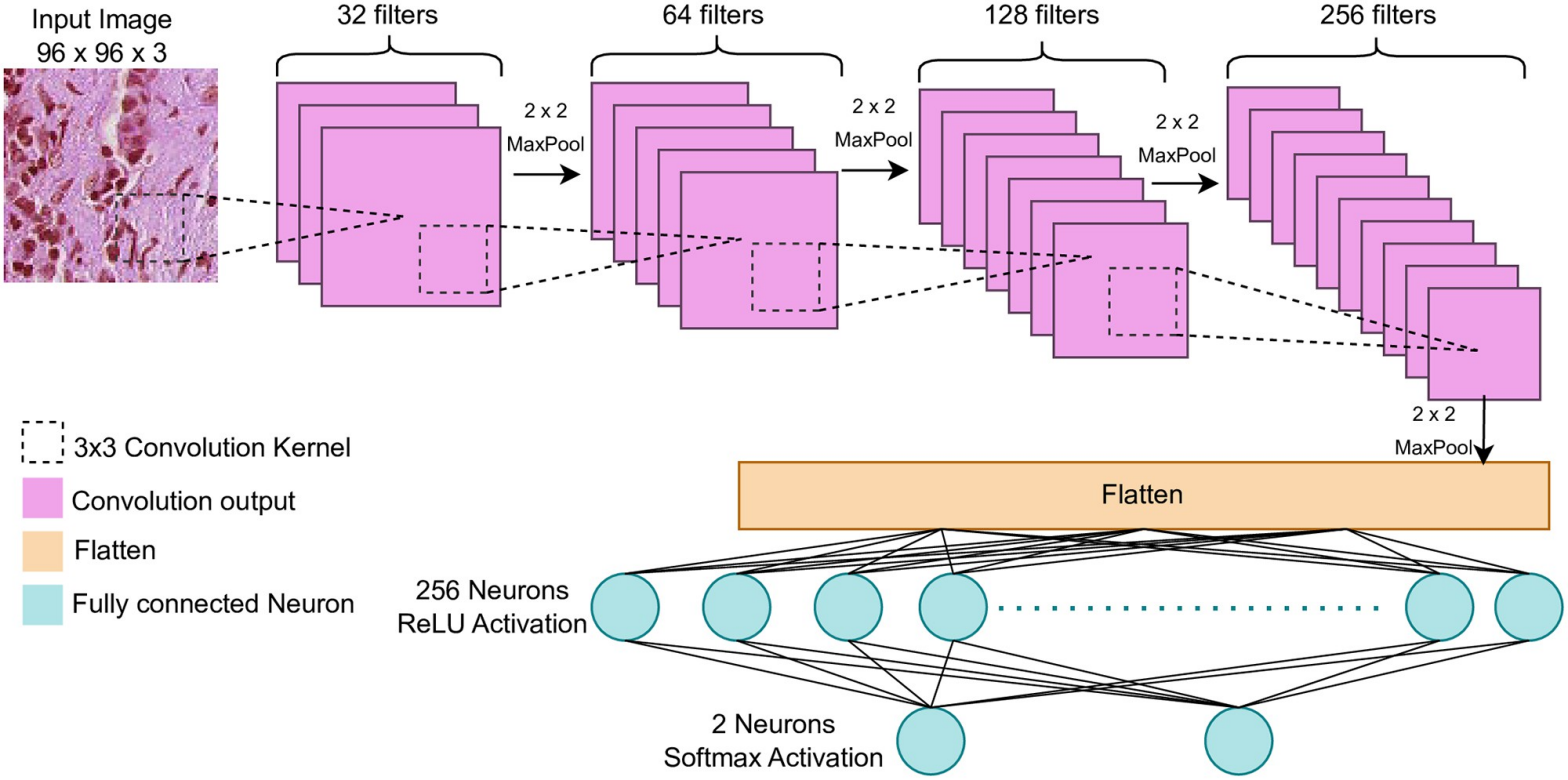
The 4-layer CNN architecture.

The student model is a rather simple lightweight model consisting of four pairs of convolution and max-pool layers in the feature extractor, alongside a Multi-layer Perceptron attached as the classifier. The MLP classifier has 256 neurons in the first fully connected layer (ReLU activation), followed by a two-neuron output layer (softmax activation). Ideally, we expect this model to not perform well on the PCam dataset due to having a small number of parameters. However, at a later stage, when training on the small MLP dataset, having a small lightweight model is necessary. The convolution layers of the model use a 3x3 convolution kernel with a stride of 1 in the X and Y axis with no padding enabled. Fig 4 depicts the architecture of the 4-layer CNN. Just like the teacher model, the student model was also compiled with categorical cross-entropy loss and Adam optimizer.

#### 3.2.3 Distillation setup

**Fig 5:**
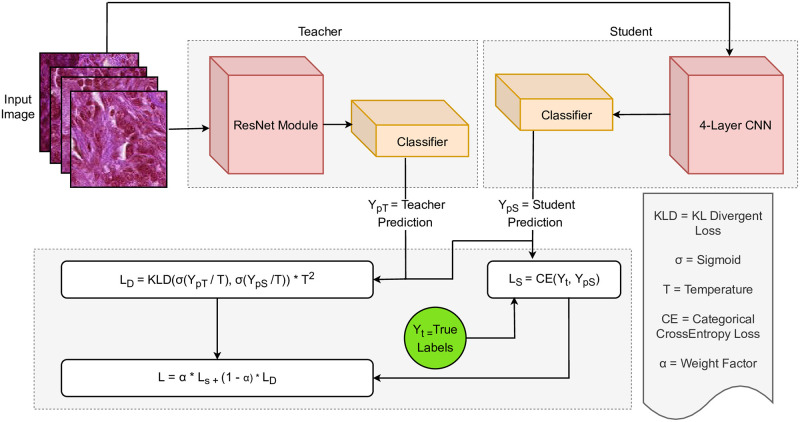
Loss calculation in the distillation environment.

As visualized in Fig 5, at the first step of the distillation, the teacher ResNet model was trained on the PCam dataset for 10 epochs, and the model with the best validation accuracy on a particular epoch was saved. Afterward, the teacher model was loaded and the student model was built so that it could learn from both the teacher and the dataset. In the distillation environment, the student generally uses a combination of distillation loss and student loss, where the distillation loss indicates the difference between the teacher’s softmax output from the student’s softmax output and the student loss indicates the error made by the student compared to the true labels. Supposedly, the pre-softmax logit from the teacher is defined by *Y*_*pT*_, and the student output labels are defined by *Y*_*pS*_. Both the values are divided by a temperature hyperparameter *T* for scaling purposes, resulting in *Y*_*pT*_/*T* and *Y*_*pS*_/*T*. Afterward, a softmax function is applied on both the scaled logits, resulting in *σ*(*Y*_*pT*_/*T*) and *σ*(*Y*_*pS*_/*T*). In the next step, a loss function is applied to calculate the loss between the softmax output of the scaled logits to calculate the distillation loss. In our case, we picked Kullback-Leibler Divergence (KL Divergence) as the function to calculate the distillation loss. Finally, the entire resultant expression is multiplied by *T*^2^ for further smoothing. Therefore, the distillation loss *L*_*D*_ stands as,

$$\begin{array}{c} L_{D}=KLD(\sigma (\frac{Y_{pT}}{T}),\sigma (\frac{Y_{pS}}{T}))*T^{2} \end{array}$$ (1)

Where,

$$\begin{array}{c} K\;L\;D\left(y_{1},y_{2}\right)=\sum_{i}y_{1}\left(i\right)*{\text{log}}_{10}(\frac{y_{1}\left(i\right)}{y_{2}\left(i\right)}) \end{array}$$ (2)

Meanwhile, the student loss *L*_*S*_ is calculated by passing the true labels *Y*_*t*_ and the student predicted labels *Y*_*pS*_ through the categorical cross-entropy loss function. The student loss is defined by,

$$\begin{array}{c} L_{S}=C\;E\left(Y_{t},Y_{pS}\right) \end{array}$$ (3)

Where, if there are n classes,

$$\begin{array}{c} C\;E\left(y_{1},y_{2}\right)=-(\sum_{i=1}^{n}y_{1i}*{\text{log}}_{10}\left(y_{2i}\right)) \end{array}$$ (4)

Finally, the total loss is obtained by combining the student loss *L*_*S*_ with the distillation loss *L*_*D*_. An additional factor *α* is utilized to weight the student loss and 1 − *α* is used to weight the distillation loss. The final loss value is defined by,

$$\begin{array}{c} L=\alpha *L_{S}+\left(1-\alpha \right)*L_{D} \end{array}$$ (5)

Or from Eqs 1 and 3, we get,

$$\begin{array}{c} L=\alpha *C\;E\left(Y_{t},Y_{pS}\right)+\left(1-\alpha \right)*K\;L\;D(\sigma (\frac{Y_{pT}}{T}),\sigma (\frac{Y_{pS}}{T}))*T^{2} \end{array}$$ (6)

Utilizing the combined loss value, the student model is trained for 10 epochs based on the teacher and the PCam dataset. The model with the best validation accuracy is saved for later usage on the MPN dataset.

### 3.3 Step 3: Student retrieval

**Fig 6:**
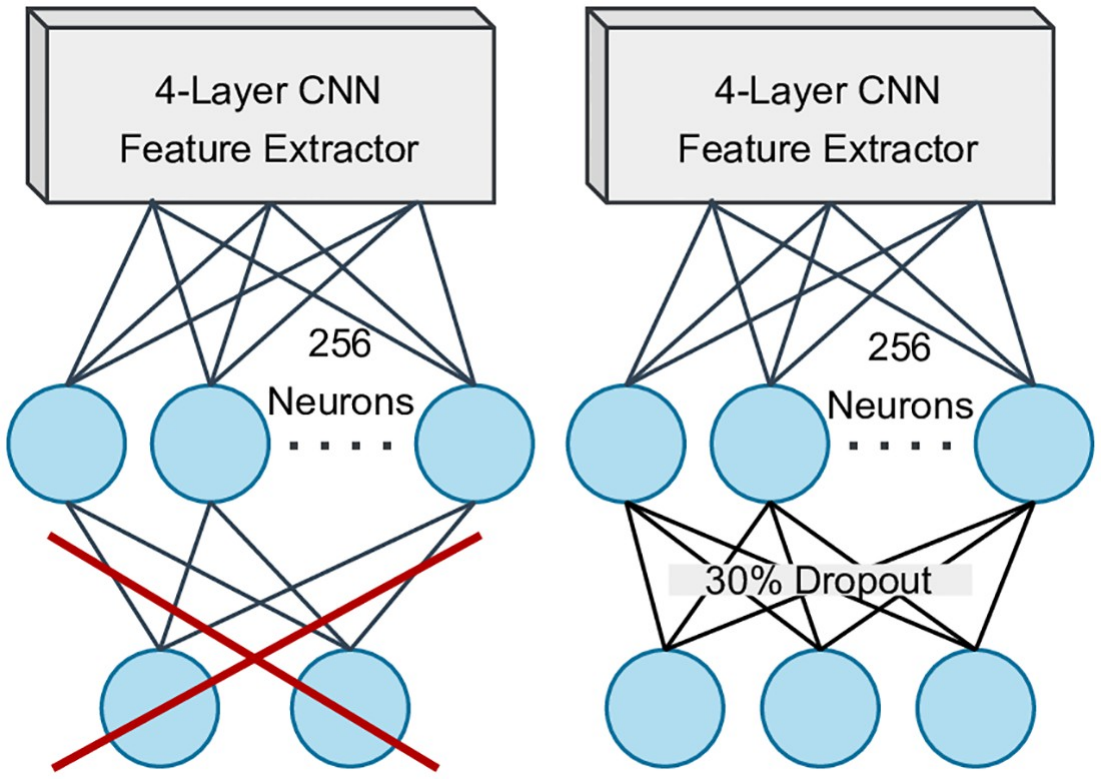
CNN model modification for training on the MPN dataset.

The saved 4-layer student CNN model from the distillation stage is loaded for further fine-tuning on the MPN dataset. However, the saved student model has two neurons in the output class, corresponding to the binary classes of the PCam dataset. The MPN dataset has three classes corresponding to the three MPN subtypes and as a result, the output layer needed to be modified to have three neurons. The changes made to the CNN model has been provided in Fig 6.

The initial output layer was replaced with a 30% dropout enabled softmax activated output layer of three neurons. With a 30% dropout rate enabled, during fine-tuned training on the MPN dataset, 30% of the randomly selected weight connections remain inactivated per epoch, resulting in fewer parameters being tuned. Dropout reduces the tendency of overfitting, which is a necessary trait required for training on the small dataset.

### 3.4 Step 4: Student training

The MPN dataset images are converted to 96x96 resolution to match up the input size of the 4-layer CNN. Finally, the modified model was trained with 5-fold stratified cross-validation setup.

#### 3.4.1 5-fold stratified cross-validation

**Fig 7:**
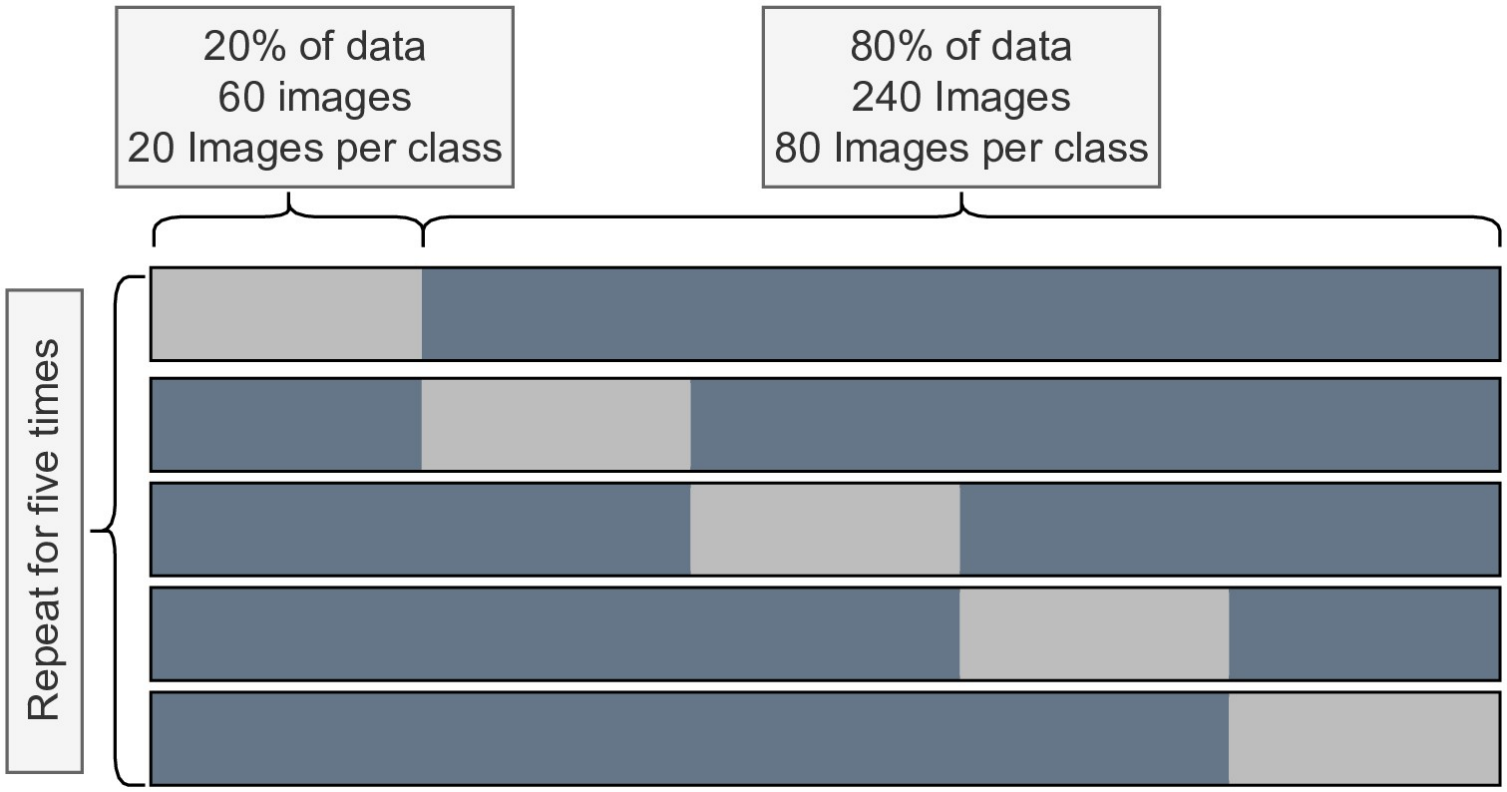
5-fold stratified cross-validation setup.

K-Fold cross-validation is usually performed to ensure that a model performs uniformly across the entire dataset. In usual cases, K = 10 is a common parameter, where 10% of the dataset goes to the test set and the rest goes to the training set, with the process being repeated 10 times across the entire dataset. However, for a dataset of only 300 images consisting of three classes, having 10-fold cross-validation ensures only 30 images in the testing set per iteration, with an average of 10 images per class. Having such a low distribution in the test set creates large swings in accuracy scores for only a few differences in misclassification volume. Therefore, as depicted in Fig 7, K = 5 was chosen for the experiment, with 20% of the dataset going into the test set and the rest going into the training set. This results in a training set of 240 images and a test set of 60 images per iteration that repeats five times.

In addition, the stratified variant of the cross-validation was applied to ensure a uniform distribution of classes according to the ratio in the actual dataset. Thus, 80 images per class were used in the train set and 20 images per class were used in the test set per iteration.

### 3.5 Step 5: Performance evaluation & comparison

To evaluate the effectiveness of the proposed method, some other reference pipelines are created. The pipelines include:

- Training the 4-layer CNN model from scratch on the MPN dataset.
- Using direct transfer learning from PCam dataset instead of transferring distilled knowledge.
- Training the 4-layer CNN model on an augmented MPN dataset. The augmentation details are given in Table 4.
- Using popular pre-trained CNNs.
- Extracting basic handcrafted features to train different ML classifiers.

**Table 4:** Processing steps during the augmentation pipeline.

Augmentation type	Details
**Rotation**	20°
**Width Shift**	20%
**Height Shift**	20%
**Flip**	Horizontal

The idea is to test other combinations of approaches as much as possible and verify whether the proposed approach fares any better against them. For comparison, we primarily extracted metrics such as accuracy, precision, recall, f1-scores, and confusion matrices.

Finally, the primary driving forces behind our methodology are:

- The PCam dataset is large and a big model is needed for proper learning from it. Thus, ResNet, a model known to perform well on large datasets, is chosen.
- Since the student model ultimately needed to be trained on a very small dataset, the model was designed to be simple and lightweight.
- Knowledge from the large teacher model, which should have more generalizability, was transferred to the student for better learning.
- The output layer of the student was modified with dropout to match the class number of the smaller dataset and to prevent overfitting.
- Other experimental pipelines are created to justify the effectiveness of the proposed approach.

## 4 Result and analysis

The training session was done on a device with the configuration of Ryzen 7 3700X 3.6GHz CPU, 16 GB RAM, and RTX2070 Super GPU. We observed the training and validation accuracy during the training phase, and in the test phase, we primarily checked test accuracy, precision, recall, and f1-Score.

### 4.1 Distillation phase on PCam dataset

The entire distillation phase involves training the teacher model on the larger dataset alongside distilling the knowledge to the student, saving the student model at the best state, and fine-tuning the student model on the smaller dataset.

#### 4.1.1 Teacher performance

**Fig 8:**
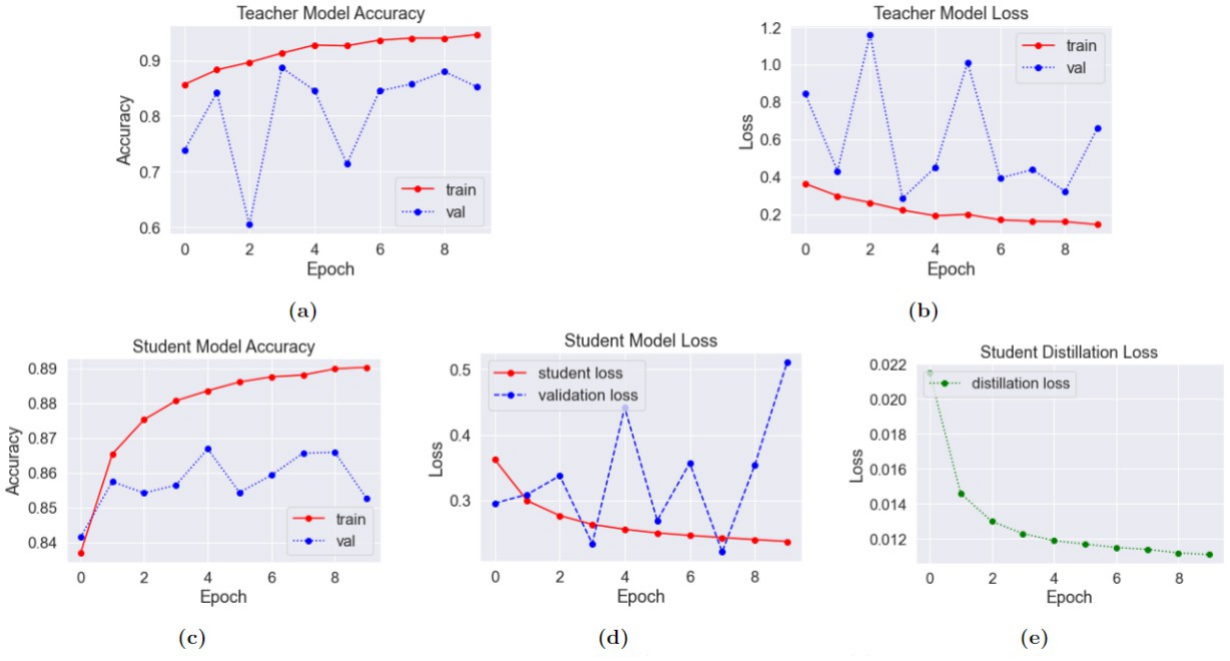
Distillation phase training graphs on PCam dataset. (a) Teacher Accuracy (b) Teacher Loss (c) Student Accuracy (d) Student Loss (e) Distillation Loss.

**Fig 9:**
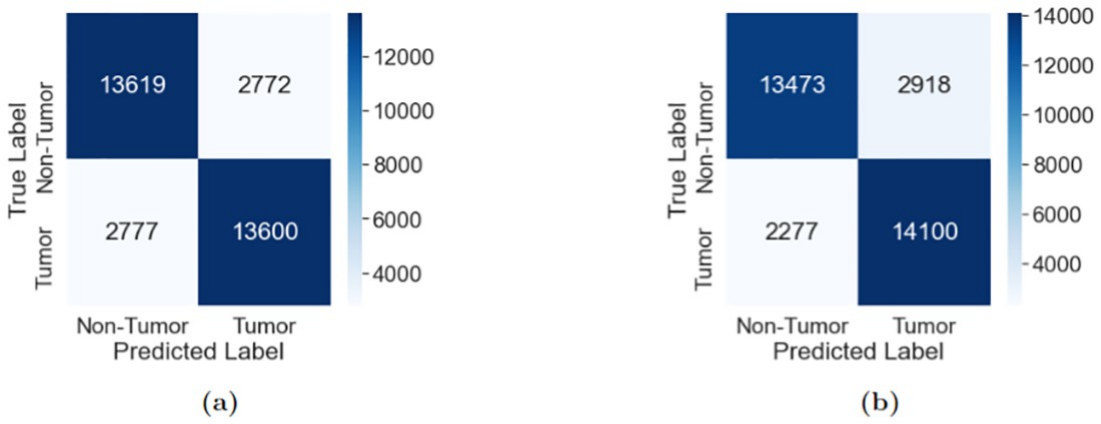
Confusion matrices of distillation phase on PCam dataset. (a) Teacher Model (b) Student Model.

In the first step, the teacher model is trained on the PCam dataset. The accuracy and loss change while training the teacher is visualized in Fig 8a and 8b. We observed that the teacher ResNet model reaches the peak point in terms of validation accuracy in the first few epochs, with a maximum validation accuracy of 88.7% at the fourth epoch. Beyond the fourth epoch, the model accuracy consistently fluctuated within the range of 80–88% but despite continuous improvement of the training accuracy, no major validation accuracy improvement was observed. Ultimately, this is a sign of the teacher model reaching an overfitted state which would only get worse with time. Therefore, the training was stopped at the 10th epoch, and the model weights for the fourth epoch were saved. The saved model achieved 83.0% accuracy on the test dataset and the confusion matrix of the result is given in Fig 9a while the classification report is given in Table 5. The teacher model achieved a precision, recall, and f1-score of 83% for all the classes across the board.

**Table 5:** Teacher (ResNet) classification report on PCam dataset.

	Precision	Recall	f1-Score
**0-Without Tumor**	0.83	0.83	0.83
**1-With Tumor**	0.83	0.83	0.83

#### 4.1.2 Student performance

The teacher model saved at the fourth epoch was further loaded and the 4-layer CNN student model was initiated. At this phase, the teacher model was necessary for calculating distillation loss, which in combination with the student loss formed the final loss value. In the distillation training phase, despite the slight continuous improvement of training accuracy throughout the 10 epochs, the validation accuracy hit the maximum at the fifth epoch, with no improvement afterward. Consequently, the student model was saved at the fifth epoch, where the student showcased the maximum validation accuracy, for further fine-tuning on the MPN dataset. The training accuracy, training loss, and the distillation loss changes are given in Fig 8. Beyond the 7th epoch, the student model showed clear signs of overfitting as the validation loss value increased rapidly despite training loss going down. Therefore, the training was stopped at the 10th epoch.

The saved student model at the fourth epoch achieved 84% accuracy on the test set, which is pretty similar to the performance of the much larger ResNet model, showcasing the power of distillation. A visualization of the confusion matrix in Fig 9b shows an improvement in classifying tumor positive images, while the performance of classifying tumor negative images decreases. The differences are generally not very large and could be attributed to random chance. Regardless, for a very small model, the 4-layer CNN showcased quite satisfactory performance on the PCam dataset. In the later phase, this student model was loaded for further fine-tuning on the MPN dataset. The classification report for the student is given in Table 6, where we can see that the student achieves better recall value for tumor-positive classes compared to the teacher, reflecting the same information as the confusion matrix given in Fig 9b. The decent performance of the student model can be greatly attributed to the better generalizability it achieved thanks to the knowledge of the teacher.

**Table 6:** Student (4-layer CNN) classification report on PCam dataset.

	Precision	Recall	f1-Score
**0-Without Tumor**	0.86	0.82	0.84
**1-With Tumor**	0.83	0.86	0.84

Training and evaluation of the student model joint with the teacher on the PCam dataset ends the distillation phase. The entire phase is a preparation for the actual training on the target MPN dataset, which is accomplished in the phase afterward.

### 4.2 Student fine-tuning on MPN dataset

**Fig 10:**
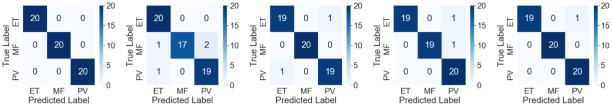
Per fold confusion matrix on MPN dataset for CNN model with distilled knowledge.

In the fine-tuning phase, the previously saved student model was loaded and trained on the MPN dataset under a 5-fold cross-validation setup. Due to having little amount of data in the training set for a small model, we found the improvement of training accuracy to be slower and therefore, the model was trained for 30 epochs for each fold. After the completion of 5-fold, average precision, recall, and f1-score across all the folds were calculated. The resultant confusion matrices and the classification report are given in Fig 10 and Table 7 respectively. We can see that the model achieved pretty consistent results across all five folds, with a maximum of one or two misclassified images per class per fold, showcasing the reliability of the results. Meanwhile, the classification report showcases the average precision, recall, and f1-scores across the folds.

**Table 7:** Student classification report.

	Precision	Recall	f1-Score
**ET**	0.97	0.97	0.97
**MF**	1.00	0.96	0.97
**PV**	0.94	0.98	0.96

The student model achieved an accuracy of 100%. 93.33%, 96.67%, 96.67%, and 98.33 across the folds, resulting in an average accuracy of 97%. From the classification report and confusion matrices, we can see that the model performed slightly better while classifying the PV images, alongside performed worst on MF images and the performance on the ET images was in between.

**Fig 11:**
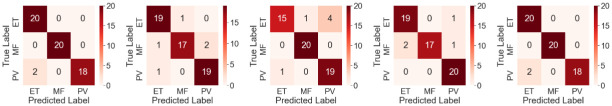
Per fold confusion matrix on MPN dataset for CNN model with transferred knowledge.

**Fig 12:**
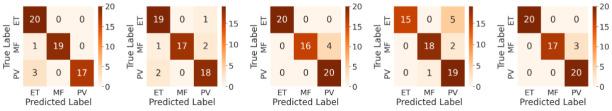
Per fold confusion matrix on MPN dataset for CNN model with augmentation.

**Fig 13:**
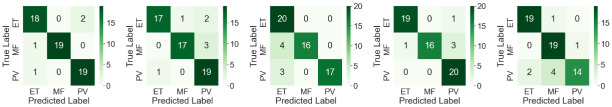
Per fold confusion matrix on MPN dataset for CNN model trained from scratch.

To further verify the effectiveness of the system, we cloned the 4-layer CNN model and created three different pipelines. One of the pipelines was about training the CNN model from scratch on the MPN dataset. The other pipeline introduced basic augmentation to train the CNN model. Finally, the third pipeline was about fine-tuning the CNN model on the MPN dataset that was previously trained on PCam dataset directly without any distillation, which is essentially a version of domain-affiliated transfer learning. The 4-layer CNN models used in the different pipelines were architecturally the same, used the same hyperparameters, and were trained for 30 epochs for each fold. In addition, 5-fold cross-validation was used and the same random seed as the distillation pipeline was set so that the folds used the identical train-test distribution across the pipelines. The proposed distilled learning mechanism showcased better performance compared to both pipelines, as can be viewed in the confusion matrices given in Figs 10–13 for all the folds. The confusion matrices of Fig 10 represent the matrics produced from the distillation pipeline, which generally shows a lower number of misclassifications compared to the other pipelines given in Figs 11–13. Additionally, the reduced number of misclassifications can be consistently observed across all the folds, which ultimately showcases the superiority of fine-tuning the distilled model instead of using basic transfer learning or training from scratch. Table 8 provides a summary of the number of misclassified samples and the differences across the pipelines.

**Table 8:** Number of misclassified samples per fold across each pipeline.

	Distillation	Transfer Learning	Augmented	Normal
**Fold 1**	0	2	4	4
**Fold 2**	4	5	6	7
**Fold 3**	2	6	4	7
**Fold 4**	2	4	8	5
**Fold 5**	1	2	3	8
**Total**	**9**	**19**	**25**	**31**

**Fig 14:**
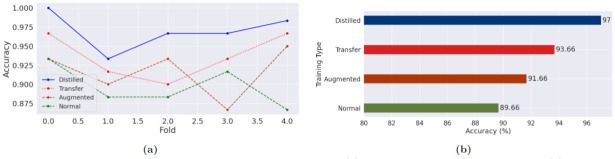
Accuracy comparison in five-fold phase on MPN dataset. (a) Per Fold Accuracy for Each Pipeline (b) Average Accuracy for Each Pipeline.

**Fig 15:**
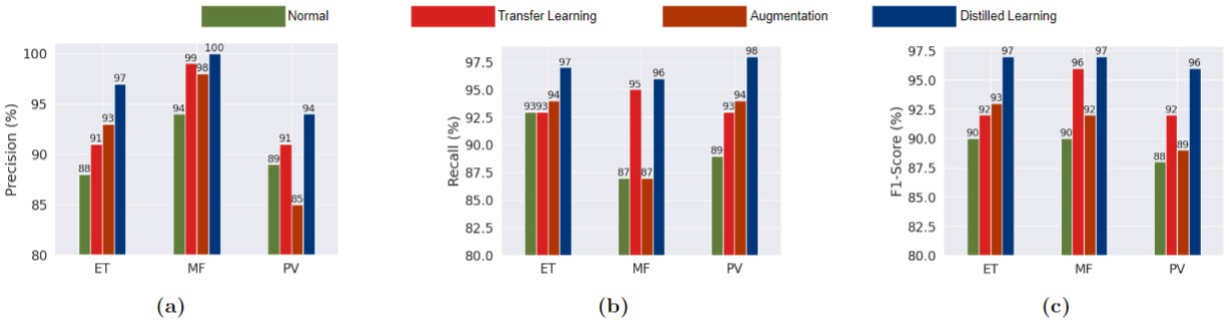
Comparison between precision, recall, and F1-score across all the pipelines in five fold phase on MPN dataset. (a) Precision (b) Recall (c) F1 Score.

A comparison between the cumulative performance of the pipelines can be better observed in Figs 14 and 15, showcasing the accuracy, precision, recall, and f1-scores. The blue colored line representing the distilled model accuracy in Fig 14a is a clear visualization of the superior performance, as suggested by the confusion matrices previously. The accuracy score never fails to be on the top of the accuracy scores produced by the other pipelines across the folds. Meanwhile, Fig 15 shows the difference between average precision, recall, and f1-scores, with all the scores being consistently higher for the distillation pipeline.

### 4.3 Comparison against other feature extraction approaches

We compared the performance of the approach against other feature extraction procedures, ranging from complex to straightforward ones, that are popular and generally work well on small datasets. In addition, we also trained some traditional machine-learning models on the raw dataset. For feature extraction, the following procedures were used:

**Histogram of Oriented Gradients (HOG)**—It is a popular feature extraction method that can be used alongside various machine learning models for classification and recognition tasks. HOG divides an image into several blocks to calculate the image gradient of those blocks across the X and Y axes. The histogram of the gradients then can be used to feed any machine-learning model for classification tasks [39].

**Prewitt Operator (PO)**—Prewitt operator is a traditional technique introduced in 1970 to extract vertical and horizontal edges from images. The algorithm primarily uses two convolution kernels, one is for extracting horizontal features and another for extracting vertical features. It is lightweight, easy to use, and can also be used in medical image-based works [40].

**Mean Pixel Value (MPV)**—It is a simple technique that calculates the average of pixel values of the entire image or at a given specific dimension. It is not usually known for being able to capture complex features but it can be useful to reduce the feature dimension. For our task, we averaged the pixel value across the third dimension, which represents the RGB channels.

Using these feature extraction procedures, we trained five machine-learning classifiers; namely Support Vector Machine (SVM), Multi-layer Perceptron (MLP), Random Forest (RF), Logistic Regression (LR), and AdaBoost (AB). We also trained these models on raw images without any feature extraction, the results of which are given in Table 9.

**Table 9:** Results of traditional ML models.

Model	Feature Extractor	Accuracy	Precision			Recall			F1-Score		
			ET	MF	PV	ET	MF	PV	ET	MF	PV
**SVM**	HOG	0.60	0.66	0.56	0.59	0.50	0.50	0.81	0.56	0.52	0.68
	MPV	0.82	0.77	0.96	0.76	0.86	0.85	0.76	0.81	0.89	0.75
	PO	0.59	0.50	0.89	0.43	0.33	0.85	0.59	0.37	0.86	0.49
	-	0.91	0.83	1.00	0.94	0.98	0.87	0.89	0.89	0.92	0.91
**MLP**	HOG	0.35	0.36	0.30	0.24	0.24	0.20	0.61	0.16	0.13	0.33
	MPV	0.42	0.53	0.31	0.49	0.11	0.76	0.39	0.17	0.44	0.37
	PO	0.36	0.38	0.35	0.34	0.26	0.75	0.37	0.30	0.39	0.35
	-	0.67	0.65	0.61	0.67	0.51	0.71	0.79	0.55	0.66	0.65
**RF**	HOG	0.53	0.48	0.52	0.59	0.29	0.48	0.85	0.35	0.49	0.69
	MPV	0.59	0.75	0.58	0.54	0.37	0.75	0.65	0.48	0.65	0.58
	PO	0.60	0.60	0.56	0.67	0.43	0.82	0.55	0.49	0.67	0.59
	-	0.77	0.80	0.85	0.70	0.75	0.78	0.80	0.77	0.80	0.74
**LR**	HOG	0.50	0.46	0.49	0.52	0.28	0.38	0.85	0.34	0.42	0.64
	MPV	0.43	0.55	0.39	0.49	0.11	0.97	0.21	0.18	0.56	0.82
	PO	0.38	0.58	0.37	0.39	0.10	0.97	0.10	0.16	0.54	0.15
	-	0.82	0.87	0.86	0.75	0.65	0.93	0.89	0.74	0.89	0.81
**AB**	HOG	0.52	0.48	0.43	0.63	0.34	0.48	0.74	0.39	0.44	0.68
	MPV	0.56	0.88	0.75	0.44	0.26	0.63	0.81	0.37	0.67	0.56
	PO	0.52	0.45	0.61	0.74	0.76	0.54	0.28	0.56	0.55	0.40
	-	0.74	0.88	0.75	0.66	0.56	0.84	0.82	0.67	0.78	0.73
**Proposed**		**0.97**	**0.97**	**1.00**	**0.94**	**0.97**	**0.96**	**0.98**	**0.97**	**0.97**	**0.96**

Table 9 showcases that the general feature extraction procedures do not work well on the MPN dataset. Every classifier achieved much lower performance on extracted features compared to raw images. Additionally, looking at the recall performance on extracted features, we can often see high recall value on one class alongside extremely low recall values on the other two, indicating that the models are not really learning anything useful and rather giving a single class as prediction output for all the images. Perhaps the general feature extractors are not suitable to extract useful information from the intricate texture of the histopathological images, hence the ML classifiers failed to properly distinguish the image classes. Meanwhile, the performance on raw images was generally better, with SVM performing the best with 91% accuracy. In general, SVM achieved better performance across the board compared to the other classifiers, with a rivaling performance to that of 4-layer CNN on the augmented dataset. Previous research claims that SVM works well on sparse and complex datasets [41] and this is a good showcase of such behavior. However, despite the decent results, the performance is still much worse compared to the distillation pipeline.

### 4.4 Comparison against pre-trained CNN models

We performed additional comparison tests against pre-trained CNN models; namely the Inception V3 [42], 50-layer ResNet [36], and MobileNet [43] model, the comparisons of which are given in Table 10. The models use the same augmentation parameters as given in Table 4. For each of the models, we used the Adam optimizer with a learning rate of 0.001 and a categorical cross-entropy loss function.

**Table 10:** Comparison against the results of the other CNN architecture and literature.

Model	Accuracy	Precision			Recall			F1-Score		
		ET	MF	PV	ET	MF	PV	ET	MF	PV
**Inception V3**	0.84	0.81	0.95	0.72	0.96	0.88	0.69	0.86	0.91	0.70
**ResNet-50**	0.86	0.84	0.87	0.90	0.80	0.94	0.84	0.77	0.89	0.86
**MobileNet**	0.93	0.89	0.99	0.94	0.98	0.93	0.89	0.93	0.95	0.91
**Literature [15]**	0.92	0.93	0.95	0.89	0.90	0.91	0.96	0.91	0.92	0.92
**Proposed**	**0.97**	**0.97**	**1.00**	**0.94**	**0.97**	**0.96**	**0.98**	**0.97**	**0.97**	**0.96**

Looking at the performance of the models, we observed the MobileNet model to be performing best, with the other two models lagging likely due to them having large amounts of trainable parameters not suited for the small MPN dataset, causing overfitting. In terms of class-specific performance, the recall value for the ET class is comparatively better than PV and MF. Freezing some of the earlier layers of the Inception and ResNet models could improve the performance in such cases. Regardless, just like in the case of the traditional ML approach, the proposed method yields better performance compared to the pre-trained approach as the pre-trained large architectures overfit the small MPN dataset.

### 4.5 Comparison against state-of-the-art literature

There is not much State-of-the-Art literature work available on deep learning-based MPN classification to compare against, even less considering that the existing work takes additional features alongside pathological samples or simply addresses weaknesses of the existing dataset they used, making them not directly comparable. In paper [15], the authors employed a 3-layer CNN structure with Adamax optimizer on an MPN dataset with an input resolution of 64x64 and a batch size of 100. We re-implemented the model with the same hyperparameter setup on the dataset of [19] with 5-fold cross-validation and achieved 92% accuracy, which is while good, still a little lower than the accuracy of the proposed approach.

### 4.6 Discussion

Due to the histopathological image datasets covering a wide spectrum of diseases, transfer learning has good potential for Ph-negative MPN classification, where a model could be trained on a large image dataset followed by being fine-tuned on a smaller MPN dataset. Nevertheless, there are disadvantages to straightforward transfer learning which uses the same model for both datasets. Using a heavyweight model on the bigger dataset, for example, may cause the model to overfit the smaller dataset significantly. Meanwhile, the model may not be able to learn much from the dataset if a lightweight model is used for both datasets. Due to the two-fold issue, knowledge distillation came into play. It allows the best features of both approaches to be used: a large model for the larger dataset and a lightweight model trained on the smaller MPN dataset.

For the distillation pipeline, a ResNet model was used as the teacher model and a 4-layer CNN model as the student model, which are customizable as long as the larger model is used as the teacher and a smaller model is used as the student. After employing the distillation pipeline, several other pipelines were created for an empirical comparison, which showed the superiority of the proposed approach over all the other pipelines with an accuracy of 97% against 93.66% accuracy with transfer learning, 91.66% accuracy on the augmented dataset, and 89.66% accuracy for training on scratch. This superior performance of the distillation pipeline can be attributed to the better generalizability of a larger model on the big PCam dataset, where the model got a general knowledge of histopathological images. This knowledge was then transferred to the smaller model through the distillation pipeline, and the student learned directly from the dataset alongside the teacher model. Even though the student model got access to a small volume of image samples during the fine-tuning phase, it mitigated the lack of information by utilizing the knowledge of the teacher, yielding superior performance over other pipelines.

Further analysis was conducted by extracting handcrafted features and training conventional machine learning models. Histopathology images have rather intricate textures and attention to fine details is necessary for accurate interpretation. In such cases, handcrafted feature extraction generally performs less effectively than deep features as long as the deep feature extractors have access to large volume of images. The case is reflected by the poor performance of the ML algorithms on the handcrafted features. Rather, training the ML models directly on the image pixels yielded better results, with the SVM model achieving an accuracy of 91%, which was yet far below the 97% accuracy achieved by the distillation pipeline. Therefore, the handcrafted feature based pipeline cannot be a replacement for the distillation pipeline.

In the next step, established CNN architectures were trained on the MPN dataset from scratch, and as expected, the models showcased signs of overfitting. The best-performing model was MobileNet with an accuracy of 93%, which has the least number of parameters. In comparison, heavier architectures like Inception and ResNet performed worse with an accuracy of 84% and 86% respectively. Regardless, the overall performance of the CNN models lagged due to overfitting issues. Finally, a reproduced model from the deep learning based literature yielded an accuracy of 92%, which was also lower than the performance exhibited by the proposed approach. As a result, the suggested methodology proved its viability in comparison to all other pipelines.

## 5 Conclusion

As discussed previously, deep learning based classification of Ph-negative MPN subtype is challenging as datasets are quite limited due to the disease being rare. Regardless, as there are large histopathological image datasets available for other diseases, knowledge from other domains can be utilized and the proposed approach showcases the effectiveness of using domain-centric distilled knowledge which is seemingly more effective than basic approaches such as transfer learning and data augmentation. We have performed extensive empirical analysis that showcased improved results compared to existing CNN architectures integrated with transfer learning and traditional ML models integrated with handcrafted features, with a performance boost of roughly 4–5% on a five-fold cross-validation setup. Although the proposed pipeline could benefit more from appropriate hyperparameter tuning and a higher resolution base dataset for knowledge distillation, there’s no doubt that the proposed approach holds a lot of promise for further advancement in MPN subtype identification. In addition, it is worth experimenting with the effects of distilled knowledge on other limited datasets of a particular disease where a larger dataset of the same modality is available.

### 5.1 Future recommendations

Although the proposed approach proved to be more effective than other comparable pipelines, the working procedure still has some gaps that can be worked on in the future:

- While the final resultant model is lightweight, the overall pipeline is rather lengthy with multi-phase training stages on different datasets. Therefore, training the pipeline can be difficult and the overall process can be more optimized.
- The quality of training is heavily reliant on the previous dataset from which knowledge is acquired, creating unavoidable issues generated from the properties of the former dataset. For example, the max resolution of images in PCam dataset is 96x96, restricting the resolution of MPN images to 96x96 too. Using higher resolution MPN images could improve performance, provided that we could. In the future, unless a dataset with a larger resolution is found, a super-resolution approach can be utilized on the PCam dataset to enhance the resolution further for training on higher resolution MPN dataset.
- There was little to no hyperparameter tweaking in the entire proposed pipeline and mostly default popular hyperparameter values were used. Hyperparameter optimization can be applied throughout the entire pipeline in the future. However, if any optimization is to take place, the process would rather be arduous due to the multi-phase process involving numerous models and datasets.

## Supporting information

S1 Data (BST)
